# Artificial Intelligence-Driven Social Media Analysis of Vaccine Hesitancy, Confidence, and Trust in Africa: A Scoping Review

**DOI:** 10.7759/cureus.106737

**Published:** 2026-04-09

**Authors:** Habeeb Abdulrauf, Abdulmalik A Lawal, Eyesiere Essien, Amarachi N Uma Mba, Prisca A Nenger, Gbemisola S Odejide, Paul Agada

**Affiliations:** 1 School of Communication, Western Michigan University, Kalamazoo, USA; 2 Department of Media Innovation and Journalism, Reynolds School of Journalism, University of Nevada, Reno, USA; 3 Department of Communication Studies, University of Miami, Coral Gables, USA; 4 Department of Communication Studies, West Virginia University, Morgantown, USA; 5 School of Journalism, Media, and Communication, Uganda Christian University, Mukono, UGA; 6 Department of Communication, North Dakota State University, Fargo, USA; 7 Department of Communication, North Carolina State University, Raleigh, USA

**Keywords:** africa, artificial intelligence, infodemic/misinformation, natural language processing, vaccine hesitancy

## Abstract

In Africa, the spread of vaccine hesitancy is associated with rapidly evolving digital narratives, but there is limited evidence on how artificial intelligence (AI) and natural language processing (NLP) have been used to study these narratives and the associated dynamics of confidence and trust. This scoping review mapped peer-reviewed primary studies applying AI, machine learning, and NLP to vaccine-related digital text in African settings. The review documented the data sources, analytical methods, validation, and stated policy or practice relevance of these studies. Using a Population-Concept-Context (PCC)-aligned search of PubMed/MEDLINE and Scopus, and following the Preferred Reporting Items for Systematic Reviews and Meta-Analyses extension for Scoping Reviews (PRISMA-ScR) reporting guidelines, 456 records were identified. After deduplication and dual independent screening, 13 studies were included. The included studies spanned North, West, East, and South Africa, with most analyses based on Twitter/X and supplemented by data from Facebook, YouTube, online news comments, and multi-platform social listening tools. Methodologically, the studies could be categorized as follows: (i) sentiment and topic modeling to track hesitancy, confidence, and trust-related attitude dynamics and risk signals during rollout; (ii) supervised classification for misinformation or fake news detection, including work on African languages and dialects; and (iii) operational social listening workflows that translate signals into response recommendations. Automation enabled near real-time monitoring and geographic or temporal segmentation across studies, but validation was commonly internal, and ethical transparency (privacy, consent, and data governance) was inconsistently reported. The potential of African AI-enabled infoveillance for targeted vaccine communication shows potential. However, there is a need for stronger context-sensitive model evaluation, multilingual coverage, and clearer integration pathways into decision-making.

## Introduction and background

Vaccine hesitancy, defined as the delay in accepting or refusing vaccination despite the availability of vaccination services, has been recognized as a complex behavioral phenomenon influenced by context, individual and group factors, and drivers specific to vaccines or programs [1]. This framing is particularly pertinent to African immunization systems, as hesitancy does not operate in isolation. Confidence issues can intersect with structural constraints and historical experiences, hindering uptake even when vaccines are available. Global monitoring highlights why confidence must be treated as a dynamic factor: a survey of 67 countries documented significant variations in perceptions of vaccine safety, importance, religious compatibility, and effectiveness, underscoring the need for ongoing monitoring of confidence [2]. Similarly, large-scale temporal modeling across 149 countries demonstrated measurable shifts in vaccine confidence between 2015 and 2019, highlighting confidence in vaccine importance as a particularly strong correlate of uptake. This emphasizes the policy value of early detection [3].

The rapid expansion of the information ecosystem during the pandemic meant that misinformation and accurate information circulated simultaneously, heightening these risks. Cross-platform analyses demonstrate that engagement patterns differ by platform and that misinformation from unreliable sources can spread widely and rapidly, resembling the characteristics of reliable sources. This complicates simple assumptions about visibility and influence [4]. In recognition that information overload can undermine protective behaviors, a World Health Organization (WHO) technical consultation proposed an infodemic management framework that prioritizes evidence-based messaging, cross-sector partnerships spanning government, communities, platforms, academia, and civil society, and the translation of knowledge into actionable guidance [5]. Behavioral evidence further underscores the importance of such approaches: susceptibility to misinformation about the virus has been linked to lower compliance with public health guidance and reduced willingness to be vaccinated, indicating that narrative environments can influence vaccine decisions [6].

In African contexts, confidence is closely linked to institutional trust and long-standing governance relationships. A multinational cross-sectional study among Arab seniors, including participants from African ‎countries (Egypt and Morocco), reported that perceived benefits, access to reliable information, and ‎prior vaccination history were associated with higher vaccine acceptance, whereas conspiracy ‎beliefs were associated with resistance [7]. During the pandemic, nationally representative telephone surveys in six sub-Saharan African countries revealed generally high levels of willingness to be vaccinated. However, safety concerns, particularly regarding potential side effects, remained the most common reservation, with pockets of hesitancy observed among certain urban and higher socioeconomic groups [8]. ‎A global map of COVID-19 vaccine acceptance rates showed marked heterogeneity across African ‎subregions, with hesitancy appearing more pronounced in West/Central Africa compared with East, ‎South, and North Africa. Further country-level evidence illustrates variability and the operational significance of trust. For example, a cross-sectional survey in the Democratic Republic of Congo reported insufficient willingness to implement strong transmission reduction measures and highlighted denial of and hesitancy toward the virus among healthcare workers as critical barriers [9].

Qualitative and applied research shows how misinformation narratives become embedded within local political and religious contexts. In Nigeria, for example, circulating conspiracy theories and misinformation have been linked to distrust of the government and religious interpretations, which shape how demand-generation communications must be framed [10]. In South Africa, vaccine hesitancy has been associated with vaccine literacy, perceptions of risk, and trust in the government's ability to safely roll out vaccinations, suggesting that communication should be combined with broader trust-building measures [11]. In Kampala, Uganda, widespread exposure to rumors about unverified adverse effects has contributed to lower uptake and reluctance, illustrating how misinformation can directly erode acceptance in urban African settings [12].

Scope of review

This scoping review maps peer-reviewed primary studies that apply artificial intelligence (AI) and natural language processing (NLP) to digital discourse in Africa on vaccines, including topics such as confidence, trust, sentiment, hesitancy, and misinformation. The review focuses on social and online textual data from platforms such as Twitter/X, Facebook, YouTube comments, online news articles, and social listening streams. These were identified through searches in PubMed/MEDLINE and Scopus and selected using the Population-Concept-Context (PCC) criteria with the Preferred Reporting Items for Systematic Reviews and Meta-Analyses extension for Scoping Reviews (PRISMA-ScR) reporting guidelines. The review summarizes the settings, platforms, vaccine topics, analytical techniques, validation, ethical considerations, and pathways through which digital analytics could inform immunization communication and decision-making support in Africa, for policy and practice purposes.

Aims and specific objectives of the review

This review aims to map and summarize the use of AI/NLP and social media analytics in the study of vaccine hesitancy-related constructs in Africa. It also aims to identify gaps in methodology, ethics, and the translation of findings into action that are relevant to immunization decision-making. Specifically, the review describes the characteristics of included African studies, including country/region coverage, platforms/data sources, vaccine focus, and dataset scale; classifies the AI/NLP technique families and operationalizations used to analyze hesitancy, trust, sentiment, and misinformation, including validation approaches and ethical reporting; and synthesizes reported outputs and articulated public-health utility pathways, highlighting evidence gaps that constrain policy integration and responsible implementation in African immunization programs.

## Review

Methods

Design and Reporting Standards

This scoping review employs the PCC framework to map the characteristics and breadth of AI-driven and NLP-based approaches to studying vaccine hesitancy and related constructs in African contexts. This study will report in accordance with the PRISMA-ScR, including a flow diagram and transparent documentation of selection decisions. No protocol registration was undertaken. In line with the scoping review's aim of classifying and describing evidence rather than estimating effects, no risk-of-bias or critical appraisal was performed. Methodological safeguards include explicit eligibility criteria, a multi-database strategy, standardized data charting, and dual screening.

Eligibility Criteria

The inclusive PCC criteria were applied to capture African vaccine-related digital discourse analyzed using AI, machine learning (ML), and NLP approaches, while excluding non-automated and non-empirical reports. A comprehensive review of the existing literature was conducted, with rigorous scrutiny applied to ensure that studies were selected strictly in accordance with predetermined PCC and study design criteria. The study incorporated peer-reviewed primary studies that applied AI, ML, and NLP methods to social or digital text data, examining vaccine hesitancy, sentiment, trust, confidence, and vaccine-relevant misinformation in African settings. Table 1 details the inclusion and exclusion criteria applied in this study.

**Table 1:** Eligibility criteria according to the PCC framework. PCC: Population-Concept-Context; AI: artificial intelligence; ML: machine learning; NLP: natural language processing.

Item	Inclusion criteria	Exclusion criteria
Population	Online public/users producing vaccine-related digital content in African settings (country-specific or Africa-subset analyses); posts/accounts/content streams geolocated to African countries or explicitly sampled from African audiences	Non-African-only datasets without Africa-specific subset reporting; non-human subjects
Concept	AI/ML/NLP-enabled analysis of vaccine hesitancy–adjacent constructs using digital text: sentiment/stance analysis, topic modeling, supervised/unsupervised text classification (e.g., misinformation), clustering-assisted sentiment, transformer-based NLP, taxonomy-based automated filtering with analytic outputs	Manual-only qualitative/content analysis without computational automation; purely descriptive statistics without automated text analytics; clinical vaccine efficacy/biomedical studies not analyzing discourse
Context	African continent; any platform generating digital trace text (e.g., Twitter/X, Facebook, YouTube comments, online news text/comments, web/social listening streams)	Non-digital contexts (e.g., clinic-only behavioral studies), unless linked to AI-based analysis of vaccine discourse data
Study designs	Primary empirical studies (observational/infodemiology, computational modeling, longitudinal trend analyses, system development + validation using African data, mixed-methods where automated analytics produce outputs)	Reviews, editorials, letters, protocols without results, conference abstracts without full text
Outcomes	Automated indicators related to hesitancy/confidence/trust/misinformation (e.g., sentiment classes, stance, topic clusters, misinformation labels, temporal/geospatial patterns, risk-tiered narratives, and dashboard outputs)	Outcomes unrelated to vaccine attitudes/information environment (e.g., general COVID-19 discourse without vaccine relevance)
Publication type	Peer-reviewed journal articles (and full peer-reviewed conference papers where applicable)	Preprints, theses, reports/guidelines, books/chapters, grey literature
Language	English	Non-English
Timeframe	2010-2025	Studies published outside this timeframe

Information Sources

The retrieval process was restricted to peer-reviewed literature indexed in two complementary databases covering interdisciplinary AI and computational social science research (Scopus) and biomedical and public health research (MEDLINE/PubMed). Searches were limited to publications from 1 January 2010 to 31 December 2025. This timeframe was selected to capture the emergence and maturation of AI, ML, and NLP applications in public health research, alongside the widespread adoption of social media platforms in African contexts during the early 2010s. Preliminary scoping indicated that African-focused studies applying automated text analytics to vaccine-related digital discourse were not published prior to 2010, while extending the search to 2025 ensured inclusion of the most recent evidence. No grey literature sources were consulted. All records were exported to Zotero, a reference management software program, for de-duplication purposes. This was followed by dual, independent screening at the title/abstract and full-text stages, with disagreements resolved through consensus.

Search Strategy

A series of database-specific strategies was developed that combined controlled vocabulary (e.g., MeSH) and free-text terms for the PCC elements ("vaccination/vaccine hesitancy"; "AI/NLP"; "social media/digital text"; "Africa"), prioritizing sensitivity over specificity. Filters were applied to restrict the results to English-language publications and peer-reviewed studies. The strategies were piloted and refined to ensure the retrieval of relevant studies across AI technique families and platforms. Table 2 shows the search string used for each database.

**Table 2:** Search strategy.

Database	Search string
MEDLINE/PubMed	((vaccin*[tiab] OR immuni?ation[tiab] OR “Vaccination”[Mesh]) AND (“vaccine hesitancy”[tiab] OR hesitan*[tiab] OR confidence[tiab] OR trust[tiab] OR misinform*[tiab] OR infodemic[tiab] OR sentiment[tiab] OR stance[tiab]) AND (“artificial intelligence”[tiab] OR “machine learning”[tiab] OR “natural language processing”[tiab] OR NLP[tiab] OR “topic model*”[tiab] OR “sentiment analysis”[tiab] OR “text mining”[tiab]) AND (Africa[tiab] OR “Sub-Saharan Africa”[tiab] OR Algeria[tiab] OR Angola[tiab] OR Benin[tiab] OR Botswana[tiab] OR “Burkina Faso”[tiab] OR Burundi[tiab] OR Cameroon[tiab] OR “Cape Verde”[tiab] OR Chad[tiab] OR Comoros[tiab] OR Congo[tiab] OR “Côte d’Ivoire”[tiab] OR Djibouti[tiab] OR Egypt[tiab] OR Eritrea[tiab] OR Eswatini[tiab] OR Ethiopia[tiab] OR Gabon[tiab] OR Gambia[tiab] OR Ghana[tiab] OR Guinea[tiab] OR Kenya[tiab] OR Lesotho[tiab] OR Liberia[tiab] OR Libya[tiab] OR Madagascar[tiab] OR Malawi[tiab] OR Mali[tiab] OR Morocco[tiab] OR Mozambique[tiab] OR Namibia[tiab] OR Niger[tiab] OR Nigeria[tiab] OR Rwanda[tiab] OR Senegal[tiab] OR “Sierra Leone”[tiab] OR Somalia[tiab] OR “South Africa”[tiab] OR Sudan[tiab] OR Tanzania[tiab] OR Tunisia[tiab] OR Uganda[tiab] OR Zambia[tiab] OR Zimbabwe[tiab])) AND (english[lang]) AND ("2010/01/01"[Date - Publication] : "2025/12/31"[Date - Publication])
Scopus	TITLE-ABS-KEY(vaccin* OR immunization OR "vaccine confidence" OR "vaccine uptake" OR "vaccine acceptance" OR hesitan*) AND TITLE-ABS-KEY("vaccine hesitancy" OR hesitan* OR confidence OR trust OR misinform* OR disinform* OR infodemic OR rumor* OR sentiment OR stance OR "risk perception") AND TITLE-ABS-KEY( "artificial intelligence" OR "machine learning" OR "deep learning" OR "natural language processing" OR NLP OR "sentiment analysis" OR "topic model*" OR "text mining" OR transformer OR BERT OR RoBERTa OR LSTM) AND TITLE-ABS-KEY(Africa OR "Sub-Saharan Africa" OR Algeria OR Angola OR Benin OR Botswana OR "Burkina Faso" OR Burundi OR Cabo Verde OR Cameroon OR "Central African Republic" OR Chad OR Comoros OR Congo OR "Democratic Republic of the Congo" OR Djibouti OR Egypt OR "Equatorial Guinea" OR Eritrea OR Eswatini OR Ethiopia OR Gabon OR Gambia OR Ghana OR Guinea OR "Guinea-Bissau" OR "Côte d’Ivoire" OR "Ivory Coast" OR Kenya OR Lesotho OR Liberia OR Libya OR Madagascar OR Malawi OR Mali OR Mauritania OR Mauritius OR Morocco OR Mozambique OR Namibia OR Niger OR Nigeria OR Rwanda OR "Sao Tome and Principe" OR Senegal OR Seychelles OR "Sierra Leone" OR Somalia OR "South Africa" OR "South Sudan" OR Sudan OR Tanzania OR Togo OR Tunisia OR Uganda OR Zambia OR Zimbabwe) AND (LIMIT-TO(LANGUAGE, "English")) AND (LIMIT-TO(DOCTYPE, "ar")) AND (PUBYEAR > 2009 AND PUBYEAR < 2026) AND (PUBYEAR > 2009 AND PUBYEAR < 2026)

Data Extraction and Synthesis

Data charting used a standardized template that was tested on 5-10 studies and then refined. The extracted variables included bibliographic details, African setting/country coverage, study characteristics, platform and trace type, vaccine-related constructs (e.g. hesitancy, trust, sentiment, and misinformation), AI/NLP technique families, specific tools and models, automated outputs and indicators, assessment and validation approaches, articulated public health or policy utility and ethical notes (where reported). The synthesis was descriptive and mapping-oriented rather than effect-estimating. A numerical summary was produced, encompassing counts by year, African sub-region/country coverage, platform, AI/NLP method family, and vaccine focus. Additionally, a narrative thematic grouping was prepared to consolidate evidence relating to: (i) the automated measurement of vaccine sentiment/attitudes; (ii) the automated detection of misinformation and infodemic signal classification; and (iii) the operational implementation of social listening/decision support and their stated pathways to communication actionability.

Results

Screening and Selection

**Figure 1:**
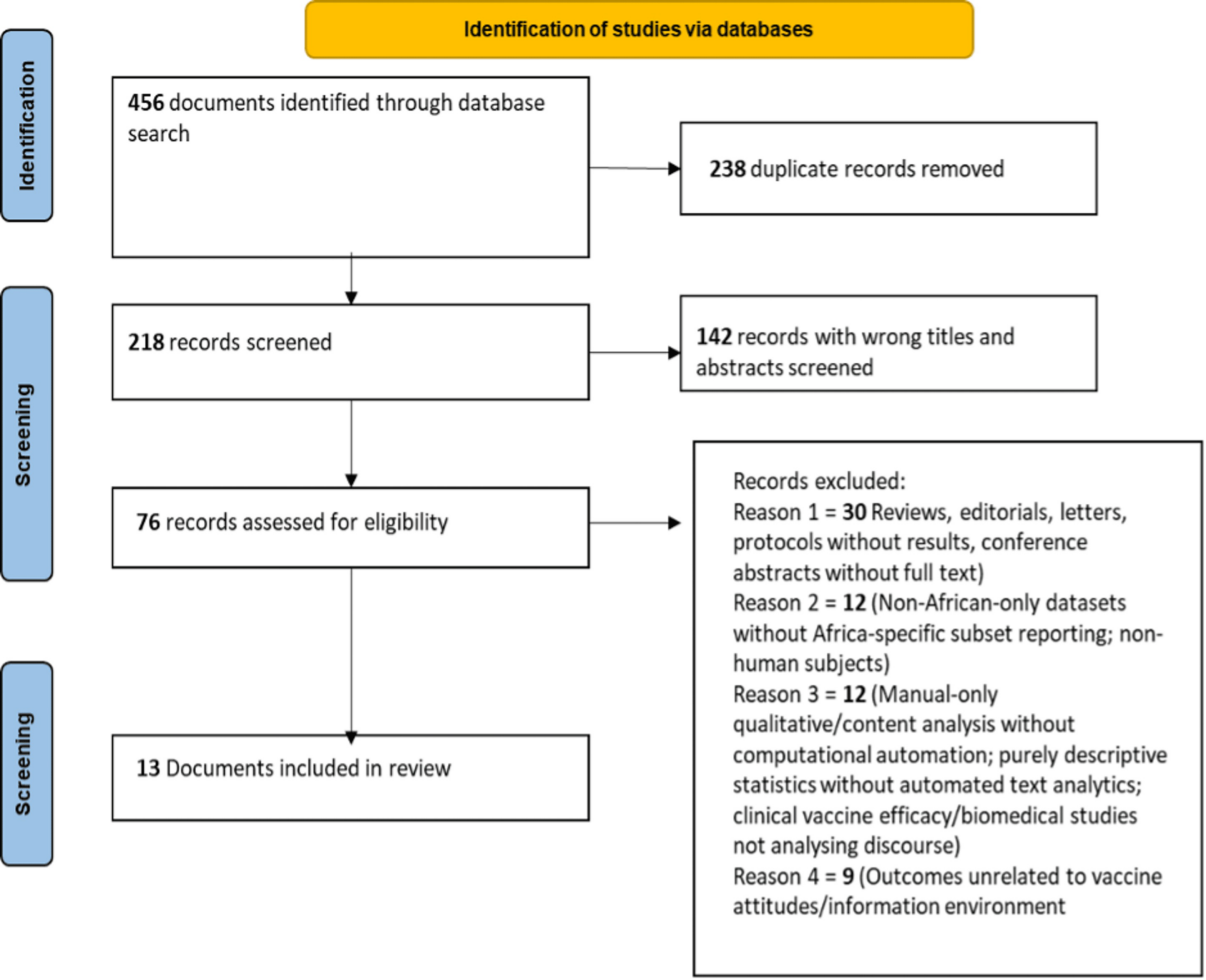
PRISMA flow diagram. PRISMA: Preferred Reporting Items for Systematic Reviews and Meta-Analyses.

The database search yielded 456 records. After removing 238 duplicates, 218 records were screened at the title-abstract level. At this stage, 142 records were excluded after title and abstract screening due to irrelevance to the predefined eligibility criteria for full-text eligibility assessment. Sixty-three records were excluded after looking at the full text. This included reviews, editorials, letters, protocols without results, and conference abstracts without full text (30 records). It also included datasets that were not only from Africa, and did not report on Africa specifically, or did not include human subjects (12 records). It also included records that only had manual qualitative or content analysis, without computational automation, purely descriptive statistics, without automated text analytics, or clinical vaccine efficacy or biomedical studies that did not analyze discourse (12 records). Finally, it excluded records with outcomes that were not related to vaccine attitudes or the information environment (nine records). Finally, 13 studies met all the criteria and were synthesized (Figure 1).

Summary of Study Characteristics

The included evidence comprises 13 Africa-focused primary studies (Table 3) that use digital text to examine vaccine sentiment, misinformation, and proxies for hesitancy. Most analyze public-facing social media platforms such as Facebook and Twitter, while others mine online newspapers, combine Twitter with news snippets, or use web-based social listening dashboards. The studies cover West Africa (Nigeria and Ghana), North Africa (Morocco and Tunisia), Southern Africa (South Africa), multi-country African datasets, and Swahili-language contexts. The dominant topic is the vaccination against the SARS-CoV-2 virus, with one study providing a pre-pandemic baseline on the broader topic of human vaccines. The methods employed range from lexicon sentiment and topic modeling (VADER, TextBlob, and latent Dirichlet allocation (LDA)) to supervised ML classifiers (support vector machine (SVM), Naïve Bayes, logistic regression, and random forest), clustering, and transformer models (BERT/RoBERTa). The scale of the data varies from small labeled corpora to tens of thousands of tweets and over one million posts. While most studies report internal performance metrics or descriptive outputs, external validation, consent, ethics, and multilingual coverage are inconsistently documented. Few papers describe linkage to routine immunization data or evaluation outcomes.

**Table 3:** Data extraction table. AI: artificial intelligence; ML: machine learning; NLP: natural language processing; DL: deep learning; MD: Moroccan Dialect; MSA: Modern Standard Arabic; LDA: latent Dirichlet allocation; MDS: multidimensional scaling; TF-IDF: term frequency-inverse document frequency; SVM: support vector machine; LR: logistic regression; NB: naïve Bayes; RF: random forest; MD-ULM: Moroccan Dialect Universal Language Model; DT: decision tree; KNN: K-nearest neighbors; LSTM: long short-term memory; BERT: bidirectional encoder representations from transformers; SBC: social and behavior change; AUC: area under the curve.

Included study (setting)	Digital platform & trace	Vaccine-related construct studied	AI/NLP technique family	Specific methods/tools reported	Automated outputs/indicators	Validation/assessment approach	Public-health/policy utility articulated
Ditsele et al. [13] (South Africa)	Twitter API v2; 21,084 tweets (Jan–Dec 2021)	COVID-19 vaccine sentiment and shifting attitudes	Lexicon sentiment classification	VADER classifier	Positive/neutral/negative sentiment trends; temporal shifts	Descriptive sentiment profiling across the rollout period	Informs strategies to promote vaccine adoption
Abiola et al. [14] (Nigeria)	Twitter; hashtag “COVID-19”; 1,048,575 tweets	COVID-19 public sentiment & topics (population risk signal; misinformation concerns)	Lexicon sentiment + topic modeling	TextBlob; VADER; LDA; MDS visualization	Polarity distribution; topic clusters	Cross-tool comparison (VADER vs. TextBlob outputs)	Supports decision-making and understanding the misinformation impacts
Gbashi et al. [15] (Africa-wide media signals)	Twitter posts + Google News headlines/snippets (Feb–May 2020)	Media polarity toward COVID-19 vaccines in Africa	Computational linguistics + neural NLP	TextBlob; VADER; Word2Vec + BiLSTM	Polarity profiles across platforms	Triangulation across 3 modeling approaches	Supports media engagement and public-health policy
Hamed et al. [16] (Tunisia)	Facebook (Tunisian users)	COVID-19 vaccine sentiment (dialect-sensitive)	Clustering-assisted sentiment classification	k-means + Naive Bayes; compared to AraBERT	Sentiment classification of Tunisian opinions	Comparative benchmarking vs. pre-trained AraBERT	Supports targeted strategies to address concerns and increase acceptance
Ashraf et al. [17] (Arabic vaccination tweets; dataset-based)	Twitter; Arcov-19Vac Arabic vaccination tweets	Vaccination misinformation	Supervised ML text classification	TF-IDF features; SVM classifier	Misinformation detection labels	Evaluated using multiple metrics on the Arcov-19Vac dataset	Supports detection/mitigation of vaccination misinformation
Madani et al. [18] (Morocco)	Twitter	COVID-19 fake news (infodemic driver affecting vaccine confidence)	NLP + ML/DL classification at scale	Apache Spark pipeline; Random Forest emphasized; feature-based classification	Fake news detection labels; contribution of sentiment as a feature	Experimental evaluation; RF accuracy reported (79%)	Supports rapid identification of misleading content during health crises
Mlawa et al. [19] (Swahili-language tweets)	Twitter; Swahili tweets	COVID-19 misinformation detection (infodemic signal relevant to trust)	Classical ML classification	LR; SVM; Bagging Ensemble; Multinomial NB; RF; Python	True vs. misinformation classification	Comparative model testing; best models reported (e.g., SVM accuracy)	Enables identification of Swahili misinformation circulating on Twitter
Ghanem et al. [20] (Morocco)	Twitter, Facebook, YouTube, + news outlet comments; 2020; MD & MSA text	COVID-19 discourse: topics + emotion/sentiment (infoveillance; trust-adjacent signal)	Topic modeling + sentiment/emotion + language model	MD-ULM built and fine-tuned; automated collection/execution platform	Dominant topics; sentiment/emotion signals; real-time monitoring	Reported outperformance vs. classical ML methods	Decision-support tool for mitigation/management via real-time infoveillance
Sommariva et al. [21] (Eastern & Southern Africa; 21 countries)	Multi-channel social listening; 300k+ posts/articles; 14M+ engagements (Dec 2020–Dec 2021)	Vaccine conversation trends + information voids + misinformation patterns	Taxonomy-based filtering + analytics + qualitative analysis	Co-developed taxonomy (9 subtopics); engagement/volume metrics + qualitative review	Subtopic trends; engagement patterns; concerns/voids/misinformation insights	Taxonomy refined with SBC teams; metric + qualitative triangulation	Guides adaptive vaccine communication and preparedness/response planning
Lohiniva et al. [22] (Ghana)	Web/social listening via Talkwalker (keyword-tracked vaccine discussions)	Misinformation identification + risk rating to address hesitancy	Social listening workflow + qualitative synthesis	Talkwalker collection; qualitative interpretation; risk assessment; knowledge co-creation	Risk-rated misinformation items + response recommendations; weekly reporting loop	Multi-sector task force review/verification of assessments	Operational infodemic management integrated into rollout decision-making
Ogbuokiri et al. [23] (Africa; nine countries)	Geotagged Twitter; 70,000 vaccine-related tweets (Dec 2020–Feb 2022)	Vaccine hesitancy hotspots inferred from sentiment geography	ML sentiment classification + spatial hotspot mapping	NB; LR; SVM; DT; KNN; hotspot technique	Hotspot maps for positive/negative/neutral sentiment locations	Reported accuracy/AUC; LR best accuracy (71%)	Supports health policy planning via geographic targeting of hesitancy signals
Sadiq et al. [24] (Nigeria)	Online newspapers (3 outlets); Dec 2020–Dec 2022	Vaccine campaign framing/tone shaping confidence	NLP + sentiment analysis on media text	Framing theory + NLP sentiment; term extraction from headlines/content	Tone profiles (headline vs. article); salient framing terms	Cross-outlet comparison	Practical guidance for crisis communication and pro-vaccine messaging
Perikli et al. [25] (South Africa)	Twitter; 30,000 tweets; hand-labeled	Vaccination hesitancy presence (sentiment classes)	Classical ML + deep learning + transformer NLP	LSTM; bi-LSTM; SVM; BERT; RoBERTa; LDA on misclassified tweets	Hesitancy-related sentiment classification; error-topic diagnostics	Model comparison using F1 (BERT/RoBERTa higher)	Improves the reliability of automated monitoring of hesitancy discourse

Thematic analysis

Theme 1: Social Media Sentiment and Topics as “Risk Signals” for Hesitancy

Most studies treat hesitancy as an inferred signal in public discourse rather than as confirmed refusal behavior. Ditsele et al. used VADER to classify 21,084 South African vaccine-related tweets and tracked shifts in positive, negative, and neutral sentiment throughout 2021 [13]. They framed sentiment as a signal for adjusting communication strategies. Abiola et al. analyzed 1,048,575 Nigerian tweets, pairing sentiment scoring with LDA topic modeling to identify their sentiment orientation for targeted response messaging and dominant concern clusters. Differences in tooling change what "hesitancy" looks like [14]. They, however, compared TextBlob and VADER outputs, demonstrating that polarity distributions can diverge for the same corpus when lexicons and scoring rules differ. Gbashi et al. compared Word2Vec+BilSTM with TextBlob and VADER, and analyzed Twitter posts and Google News snippets [15]. They suggested that platform ecology and media amplification co-produce polarity signals, which makes cross-study synthesis more difficult. Although several studies are moving toward context-sensitive modeling, validation remains mostly internal [16], for example, combining naïve Bayes with k-means clustering for Tunisian Facebook discourse, benchmarking their approach against AraBERT, and emphasizing the importance of dialect-sensitive tuning. Across studies, authors increasingly emphasized context-sensitive modeling choices and error analysis to improve interpretability, particularly in multilingual and dialect-rich settings. When it comes to these pipelines, the best approach is to view them as directional surveillance indicators rather than prevalence estimates. In order to get a more accurate picture, they should be triangulated with other indicators.

Theme 2: Misinformation Detection and Infoveillance for Infodemic Control

Several studies treat misinformation as a distinct classification task rather than a by-product of sentiment analysis. For example, Ashraf et al. developed an automated misinformation detector for Arabic vaccination tweets using term frequency-inverse document frequency (TF-IDF) features and multiple classifiers [17]. This approach positions automated filtering as a preliminary triage process that can identify the most prevalent misinformation themes. Madani et al. analyzed Moroccan social media posts, implementing an Apache Spark pipeline to classify vaccine-related misinformation and reporting an accuracy of around 79% for a random forest model [18]. Mlawa et al. curated a Swahili dataset of 1,763 tweets containing misinformation about the SARS-CoV-2 virus and compared the performance of logistic regression, SVM, bagging ensemble models, multinomial naïve Bayes, and random forest [19]. They demonstrated that benchmarking is feasible in lower-resource language settings when annotation is possible. Ghanem et al. progressed from evaluating a single model to developing a near-real-time monitoring platform in Morocco [20]. They compared deep learning and classical ML approaches and described a decision-support tool for tracking misinformation. Sommariva et al. scaled infoveillance across 21 African countries by combining taxonomy-based filtering with NLP and network analytics [21]. They tracked over 300,000 posts and 14 million engagements and iteratively refined the taxonomy with stakeholders.

Theme 3: From Analytics to Communication Decisions and Geographic Targeting

Only a subset of studies explicitly links monitoring outputs to programmatic action. Lohiniva et al., for example, operationalized Ghana’s management of the infodemic surrounding the country's response to the pandemic by using Talkwalker searches with sentiment classification and automated topic, and then feeding situation reports into immunization program teams [22]. The study documented the verification of misinformation by the task force and the development of response recommendations, moving beyond passive descriptive surveillance.

A second translation pathway is geographic targeting. Ogbuokiri et al. used geotagged Twitter data from nine African countries, applying ML sentiment classification (naïve Bayes) alongside spatial analysis to map and detect vaccine-hesitancy hotspots [23]. They reported a predictive performance of 71% accuracy and 85% area under the curve (AUC). This approach treats AI outputs as a form of triage, not to "prove" who is hesitant, but to highlight where competing narratives may cluster and where tailored communication can be prioritized, particularly when resources for in-person engagement are limited. In another analysis of Nigerian online newspapers using automated text analysis to characterize the tone and sentiment of vaccine-related messaging [24], they positioned the results as evidence to strengthen news-based communication strategies. However, across these translation-oriented studies, the downstream effects on uptake or trust are rarely evaluated, so "impact" is typically inferred from informational outputs. Lohiniva et al. demonstrate the feasibility of closing the loop from detection to response [22]. However, few studies quantify changes in uptake or trust in routine immunization contexts over time, nor do they report implementation fidelity and costs.

Africa-specific sociotechnical dimensions shaping AI/NLP evidence on vaccine hesitancy

Study and Data-Source Landscape (Digital Traces and Settings)

Across the 13 included African-focused primary pieces of research (Table 3 ), the majority of analyses depend on publicly available social media text, particularly Twitter/X, while further sources consist of Facebook discussion, news outlets' comment sections, multi-channel social listening feeds, YouTube, and online newspaper articles. Geographic coverage spans West Africa (e.g., Ghana and Nigeria), North Africa (e.g., Tunisia and Morocco), Southern Africa (e.g., South Africa), a Swahili-language setting, and multi-country African datasets. The scale of the data varies substantially, from thousands of tweets/posts to over one million tweets in a single-country corpus. For instance, Abiola et al. analyzed 1,048,575 tweets from Nigeria [14], Ditsele et al. analyzed 21,084 tweets from South Africa in 2021 [13], Ogbuokiri et al. used 70,000 geotagged tweets from nine African countries [23], and Sommariva et al. analyzed over 300,000 posts and articles linked to over 14 million engagements from 21 countries [21]. The focus on vaccines is dominated by SARS-CoV-2 vaccination discourse, alongside at least one study framed as a broader "human vaccines" discourse.

Analytic Approaches and Model Validation Patterns

In terms of methodology, the included studies cover topic modeling, lexicon-based sentiment classification, supervised ML for text classification, transformer/deep learning approaches, and clustering-assisted sentiment pipelines. Lexicon-based sentiment and topic modeling approaches make use of TextBlob, VADER, and LDA [13,14]. Comparative and hybrid pipelines are also present. For example, Gbashi et al. used TextBlob, VADER, and Word2Vec+BiLSTM to triangulate polarity across Twitter and Google News snippets [15]. Supervised classification is used for detecting misinformation and fake news. Common methods include TF-IDF and SVM [17], random forest [18], and various traditional ML models [19]. Research addressing dialect and language focuses on Hamed et al. [16], who studied Tunisian Facebook sentiment. They combined k-means with naïve Bayes and compared their results to AraBERT. Ghanem et al. also looked at Moroccan Dialect and Modern Standard Arabic modeling [20]. They used a fine-tuned language model within an automated collection and execution platform. Validation and assessment are often reported through internal descriptive profiling, comparisons between tools, or experimental evaluations using standard metrics. Fewer studies mention external validation or ties to routine immunization indicators. Assessments often include comparisons of tools, such as VADER and TextBlob in Abiola et al. [14], model comparisons showing better performance in transformer variants in Perikli et al. [25], and comparative model testing in Mlawa et al. [19].

Automated Outputs and Indicators Produced

The main outputs from these studies include trends over time, topic clusters, sentiment distributions, misinformation labels, and metrics related to engagement and volume in social listening streams. Researchers sometimes view "hesitancy" as a signal in conversations indicating refusal, instead of focusing directly on what is being refused. In these cases, automated outputs serve as surveillance indicators, such as sentiment classes (positive, neutral, or negative) tracked over time [13] or sentiment combined with topic modeling to highlight key areas of concern [14]. Variations in tools can create different sentiment distributions on the same data, as shown by Abiola et al. [14] in their comparison of VADER and TextBlob. This emphasizes that outputs depend on the methods used. In studies focused on misinformation, outputs typically consist of binary or multi-class labels that help with sorting. Examples include misinformation detection labels in Ashraf et al. [17], fake news detection labels in Madani et al. [18], and using "true versus misinformation" classification in Mlawa et al. [19]. In some cases, the outputs support near-real-time monitoring, e.g., real-time monitoring and decision support in Morocco has been indicated in Ghanem et al. [20]. Multi-channel social listening also produces engagement patterns and subtopic trend signals that are refined iteratively with stakeholders [21].

Public-Health Utility and Translation Pathways Described

Some studies clearly show how analytics can guide immunization communication and decision-making instead of just outlining them. One way this happens is through the operational management of infodemics. Lohiniva et al. describe a workflow using Talkwalker that generates response recommendations and rates misinformation items by risk [22]. This workflow is backed by verification from a multi-sector task force and involves a weekly reporting cycle. Another way to translate this is through geographical targeting. Ogbuokiri et al. combine ML-based sentiment classification with spatial hotspot mapping to find potential "hesitancy hotspot" areas from geotagged tweets [23]. They frame their outputs as a triage signal for prioritizing tailored communication where resources are limited.

Media-text analysis is also used to provide communication guidance. For example, Sadiq et al. use automated text analysis of Nigerian online newspapers to characterize the tone and sentiment of vaccine-related messaging [24]. They also suggest ways to improve news-based communication strategies. Analyzing media texts can offer guidance in communication. For instance, they also used automated text analysis of Nigerian online newspapers to identify the sentiments and tone of vaccine-related messages and to suggest strategies for news-based communication. However, even when studies outline detection-to-response mechanisms or targeting logic, they seldom evaluate the effects on uptake or trust in the available evidence. Instead, "impact" is usually inferred from feasibility demonstrations and informational outputs rather than assessed through measured behavioral outcomes.

Discussion

Summary of Key Findings

Across studies, online discourse about vaccines is heterogeneous: sentiment signals are typically mixed, varying by platform and method, and shifting with rollout events. Therefore, "hesitancy" is best treated as a surveillance proxy rather than a measured behavior. Negative narratives tend to focus on safety, distrust of institutions, adverse effects, and conspiracy or rights-based framing, whereas positive content emphasizes protection, collective benefit, and efficacy. Topic modeling and error analysis suggest that misclassification often occurs around disputed subtopics, highlighting the need for locally adapted taxonomies. Studies on the detection of misinformation show that automated triage is feasible in African-language settings, but performance reporting is inconsistent. Several studies extend analytics beyond description. For example, geotagged sentiment mapping can highlight potential hotspots for targeted outreach, while social listening workflows can illustrate how dashboards can inform verification and response cycles. However, few studies assess the ethical transparency and downstream impact on trust or uptake, and representativeness remains a persistent issue. Dialect-aware modeling shows promise but is often lacking in cross-country validation.

Comparison of Findings With Global Literature

The three resulting themes demonstrate that, in the African context, digital text can be a reliable indicator of vaccine confidence, although the interpretation of these signals remains a subject of debate. A 10-year study of human papillomavirus (HPV)-related tweets shows that polarity becomes more actionable when linked to phrase associations and entities that tie narratives to institutions and events [26]. Similarly, global Twitter work on the topic of the coronavirus vaccine likewise demonstrates event-driven shifts in topics and emotional dynamics, with "trust" emerging as a signal [27]. Aspect-based sentiment analysis provides more detail by distinguishing attitudes toward distribution, side effects, and discussions of identity [28]. However, several African studies in this review still rely on lexicons or single-task classifiers, meaning cross-setting comparisons remain vulnerable to platform ecology, sarcasm, and dialect. Cross-platform analyses confirm that sentiment can differ between Reddit and Twitter, even when trends co-move [29]. Infodemiology approaches that fuse network structure with unsupervised NLP offer a stronger template for interpreting these signals [30].

Regarding the relationship between misinformation and its translation into action, the global literature frames influence as both measurable and structural. Facebook network mapping shows that anti-vaccination groups can become embedded within undecided groups, thereby amplifying their reach through connectivity rather than size [31]. Evidence from multiple countries links online organizing and foreign disinformation to safety concerns and falling vaccination rates, highlighting the importance of governance and information security in building confidence [32]. Experiments demonstrate that exposure to misinformation diminishes the intention to vaccinate, underscoring the importance of rapid detection and counter-messaging [33]. Studies of coordinated manipulation identify recurring themes. These include personal danger, conspiracies, and civil liberties. Automated classifiers can prioritize these themes [34]. Evidence from YouTube indicates that misinformation can reduce trust among the unvaccinated, while verification searches can improve attitudes [35]. A comparison of 192 countries links mentions of adverse events and negative emotions to vaccination rates, supporting the monitoring of emotions in coverage analytics [36]. Time-lagged modeling shows that sentiment can predict acceptance the following day [37]. Geospatial time-series NLP demonstrates place-based variability that can inform targeted outreach [38]. Deep learning that maps posts to health belief model (HBM)/theory of planned behavior (TPB) constructs provides more intervention-ready segmentation than polarity alone [39]. Large-scale Twitter sentiment tracking can also detect bots and political activism within opposition content [40]. Cross-platform qualitative analysis shows that HPV misinformation narratives vary across platforms, reinforcing the need for tailored responses [41]. Real-time dashboards that operationalize sentiment and the WHO's 3Cs of vaccine hesitancy demonstrate the approach's feasibility, while highlighting the need for validation [42].

Implications for Policy and Practice

Policy: It is proposed that AI-enabled infodemic surveillance and vaccine confidence monitoring be established as a public health standard commissioned across immunization programs in African settings. This would involve routine social listening across major platforms (e.g., Twitter/X, Facebook, WhatsApp-adjacent public channels, and YouTube, where feasible), governed by ethical standards, with a minimum level of analytical capability for sentiment detection, topic identification, and misinformation triage. This would be supported by safeguards to ensure equity, taking into account digital exclusion and language diversity. A core requirement should be the adoption of validated, context-adapted NLP models for priority African languages and dialects, with transparent reporting of data provenance, bias checks, performance drift, and annotation rules. There should be a standing multi-stakeholder "infodemic response unit" embedded in Expanded Programme on Immunization (EPI) structures to review weekly dashboards, verify high-risk narratives, and authorize response actions. Standard operating procedures should include (i) hesitancy signals and risk-tiering of misinformation, (ii) predefined response playbooks aligned to vaccine schedules, and (iii) integration with service delivery intelligence (e.g., stock levels, access barriers, adverse event communication, and rumor hotlines). Quality indicators should include the time taken to detect emerging narratives, the time taken to respond, the proportion of signals verified, the documentation of corrective actions, and language coverage. In settings with limited resources, it is essential to prioritize low-cost pipelines (lexicon and lightweight classifiers where appropriate), partnerships with telecom companies and shared regional model libraries, media regulators, and fact-checking networks, while safeguarding civil liberties and avoiding punitive surveillance.

Practice: Implementing a detect-triage-respond-evaluate model is essential. Frontline teams should create dashboards that categorize signals by platform, geography, and content type (e.g., safety, side effects, conspiracy theories, and trust in institutions, access/inequity), with clear escalation thresholds for high-reach misinformation. The focus of communication practice should shift from providing generic reassurance to ensuring message precision. This involves using tailored counter-narratives that address emotional drivers and dominant clusters of concern. Digital responses should be paired with offline service corrections where access complaints are prominent. Human-in-the-loop workflows should be used, with public health experts validating automatically surfaced topics, refining labels, and approving final messages before deployment. To strengthen uptake and equity, teams should receive training in rapid rumor interviewing, culturally adapted framing, and safe engagement on digital channels, including referral pathways to trusted community voices. Outcomes should be monitored using lagging indicators (appointment completion rates, district coverage, and missed-dose rates) and leading indicators (reach, engagement quality, sentiment recovery, and rumor recurrence), with the results fed into iterative improvement cycles. Conversely, unverified amplification of misinformation, ad hoc messaging, deployment of opaque models without bias checks, and a lack of community oversight should be avoided.

Limitations of the Review

The scope of this review was limited by searching only PubMed and Scopus, restricting the search to English-language peer-reviewed articles, and excluding grey literature. No critical appraisal was performed, and heterogeneous methods prevented estimation of effects. The findings rely on platform-skewed digital traces, which often proxy hesitancy, and there is limited multilingual coverage, ethics reporting, and external validation.

## Conclusions

In African contexts, the analysis of digital discourse using AI is increasingly applied for investigating vaccine confidence, misinformation, and related signals of hesitancy. This scoping review mapped 13 primary studies that used NLP, ML, and social listening to analyze social media, news, and other digital texts. The evidence suggests that these approaches have been used to detect evolving concerns, identify dominant narratives, and support the development of targeted communication strategies by geography and over time. However, hesitancy is often measured using proxies; the models are not always validated, and coverage of African languages and dialects remains limited. Ethical transparency, evaluation of the impact on public health, and governance are inconsistently reported. Future research should prioritize the use of context-adapted multilingual datasets, external validation, open reporting standards, and the integration of analytics into immunization decision-making workflows with measurable outcomes to enable accountable public health action.
